# Assessing Postural Stability in Gastrointestinal Endoscopic Procedures with a Belt-like Endoscope Holder Using a MoCap Camera System

**DOI:** 10.3390/jpm14121132

**Published:** 2024-11-30

**Authors:** Tadej Durič, Jan Hejda, Petr Volf, Marek Sokol, Patrik Kutílek, Jan Hajer

**Affiliations:** 1 3rd Medical Faculty, Charles University, Ruska 2411, 100 00 Prague, Czech Republic; 2 Faculty Biomedical Engineering, Czech Technical University, Namesti Sitna 3105, 272 01 Kladno, Czech Republic; 3 Department of Internal Medicine, Kralovske Vinohrady Universital Hospital, Šrobarova 1150/50, 100 00 Prague, Czech Republic

**Keywords:** musculoskeletal injury, GI endoscopy, prevention, postural stability

## Abstract

**Background/Objectives:** As musculoskeletal injuries in gastroenterologists related to the performance of endoscopic procedures are on the rise, solutions and new approaches are needed to prevent these undesired outcomes. In our study, we evaluated an approach to ergonomic challenges in the form of a belt-like endoscope holder designed to redistribute the weight of the endoscope across the whole body of the practitioner. The aim of the study was to determine how the use of this holder affected the body posture of practitioners during endoscopy. **Methods:** We designed a special endoscopic model that emulates basic endoscopic movement and maneuvers. With the use of the MoCap camera system, we recorded experienced endoscopists exercising a standardized set of tasks with and without the holder. **Results:** Following video and statistical analyses, the most significant differences were observed in the position of the left arm which pointed to a more relaxed arm position. **Conclusions:** The ergonomic benefits of the belt holder in this model merit testing in the clinical setting to evaluate its effectiveness and prevention of musculoskeletal injuries in GI endoscopy.

## 1. Introduction

Gastrointestinal (GI) endoscopy is becoming one of the leading preoccupations in the everyday gastroenterology practice. Therapeutic endoscopy is on the rise, and a shift away from diagnostic endoscopy is visible, which causes a significant workload for the GI practitioner. As a result of this change, musculoskeletal (MSK) injuries among endoscopists are on the rise. A systematic review by Young et al. that included 13 studies demonstrated that the majority of interviewed endoscopists reported pain or injuries related to endoscopy. Common areas experiencing pain were the back (15–57%), neck (9–46%), shoulders (9–19%), elbows (8–15%) and hands/fingers (14–82%). In this process, several risk factors were included (procedure volume, time spent performing endoscopy, time spent in clinical practice, and age). Due to how endoscopy is performed, several experimental studies implicated a high probability of occupational injury among endoscopists. A difference in reported areas of pain between beginner and experienced endoscopists was noted, implying different mechanisms of injury [1].

Much thought has been given to injury prevention in this field. Recommendations on endoscopic room layout, body posture, time, procedure management, and so on have been published by the American Society for Gastrointestinal Endoscopy (ASGE) [2]; however, there remains a lack of technical solutions for tackling this ergonomic challenge. In 2004, a belt-like endoscope holder (ScopeDoc, COOK Medical) was conceptualized by Dr. Binmoeller. The idea of “hands-free” endoscopy was implemented, with the aim of reducing the stress and fatigue of the practitioner. Without the holder, the practitioner had to carry and steer the full weight of the endoscope, which resulted in fatigue, stress, and pain in the upper extremities. With the holder, the weight of the endoscope was transferred to the practitioner’s body [3]. The design of the device was subsequently developed further (Endojoystick, XGLU s.r.o.) to offer more maneuvering options. A study on MSK injury prevention in GI endoscopists explored the applicability of this holder in a study designed to measure the EMG (electromyography) potentials of muscles in the upper body and extremities during a simulation of endoscopic procedures. Participants performed a simulation of endoscopic movement in which the endoscope was supported on a specially designed endoscopic exercise platform. During the simulation, they wore a special suit with surface EMG sensors. Each participant performed the exercises twice: first without the holder, and for a second time using the holder. Muscle potentials and levels of microbreaks were both recorded. The results showed statistically significant differences in the observed parameters and implied that the use of ergonomic accessories can play a role in MSK injury prevention [3].

As postural stability and body posture play an important part in injury prevention, it was our intention to evaluate postural stability and body posture when using the belt-like endoscope holder.

## 2. Materials and Methods

### 2.1. Participants

As our aim was to assess postural stability in endoscopic procedures conducted both with and without the use of a belt-like endoscope holder, we recruited 4 experienced endoscopists as participants, including 3 male and 1 female endoscopists, whose ages ranged from 37 to 53 years. The participants typically performed diagnostic and therapeutic procedures using endoscopy on a daily basis. All participants were volunteers, and signed consent forms were obtained. Including the preparation of all necessary materials, the experimental room, cameras, and personal equipment, this study took approx. 3 months, and it was approved by the local ethics committee. By design, this is an experimental research study.

### 2.2. Belt-like Endoscope Holder

**Figure 1:**
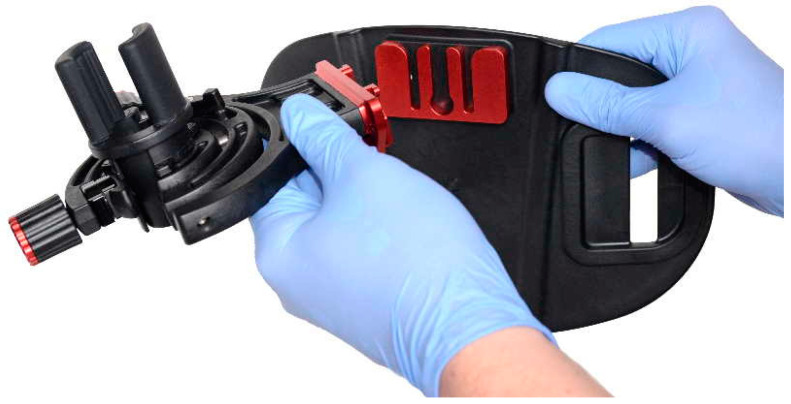
Original components of the belt-like endoscope holder (Endojoystick, XGLU s.r.o.).

The belt-like endoscope holder is essentially a belt with a central piece attached that is made up of 2 parts: a frame that allows movement of an endoscope when it is docked inside the frame, and a frame-compatible central endoscope holder that is mounted on the endoscope. All of these parts are shown in Figure 1.

**Figure 2:**
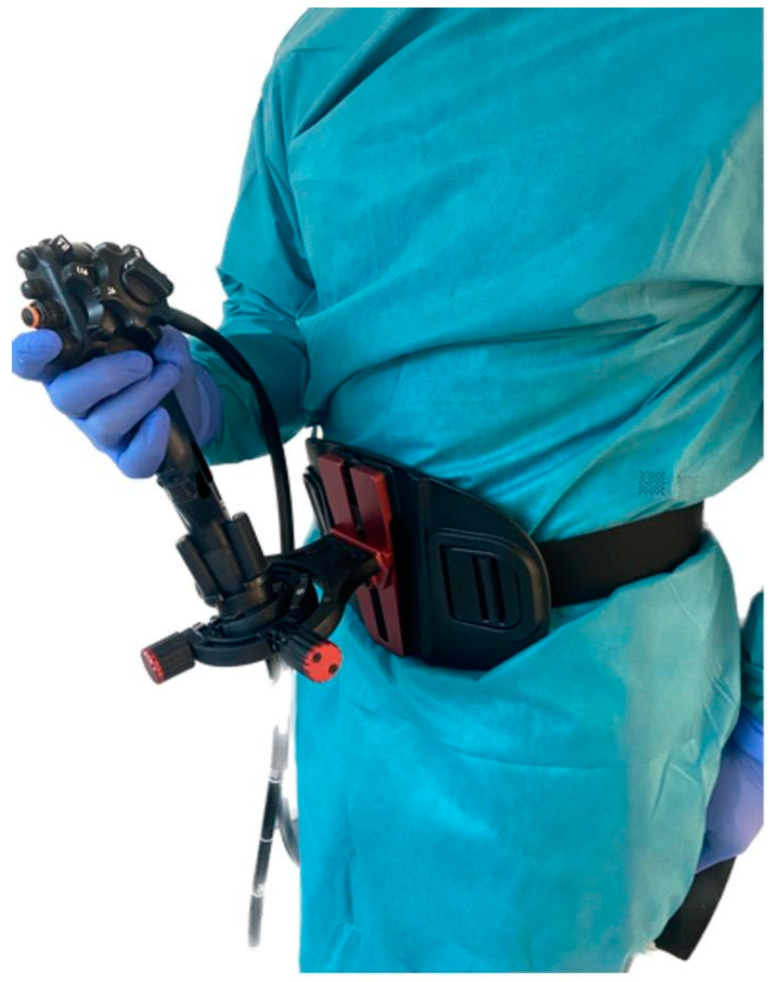
A person holding an endoscope inserted into the belt-like endoscope holder.

Figure 2 shows a person using the combination of a belt-like endoscope holder and an endoscope. The endoscope is mounted inside the endoscope holder, which is then inserted inside a central frame that has joystick capabilities. The central frame is attached to the belt holder that is attached to the endoscopist’s body.

### 2.3. Development of Standardized Procedure, Training Box Design, and Manufacturing for Task Conceptualization

**Figure 3:**
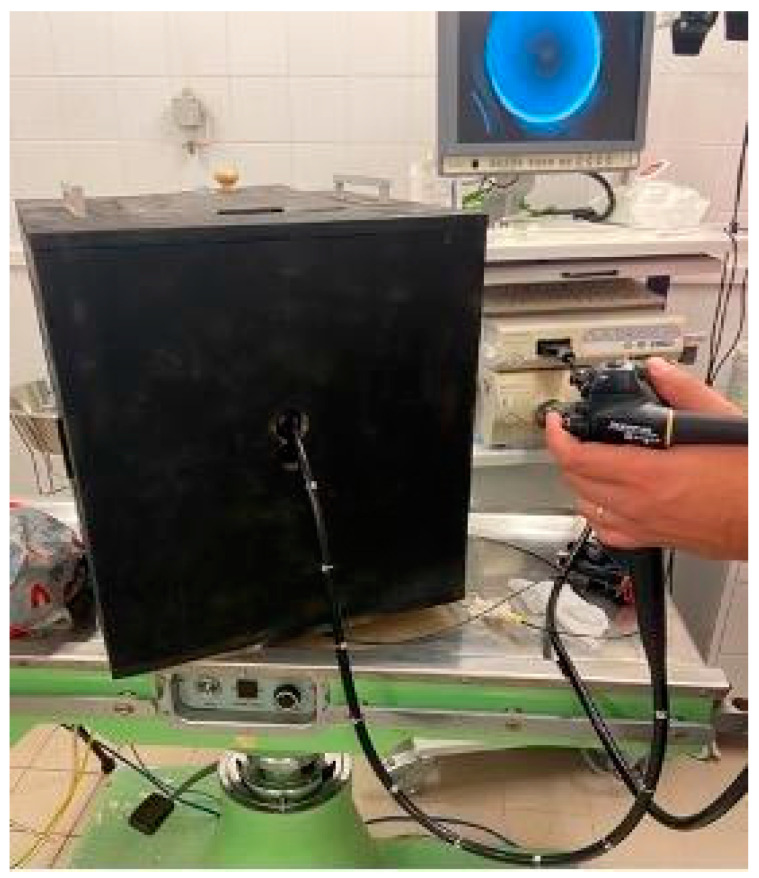
Frontal view of the training box with an endoscope inserted. The training box is placed on an experimental table; an endoscopy unit is visible behind it.

**Figure 4:**
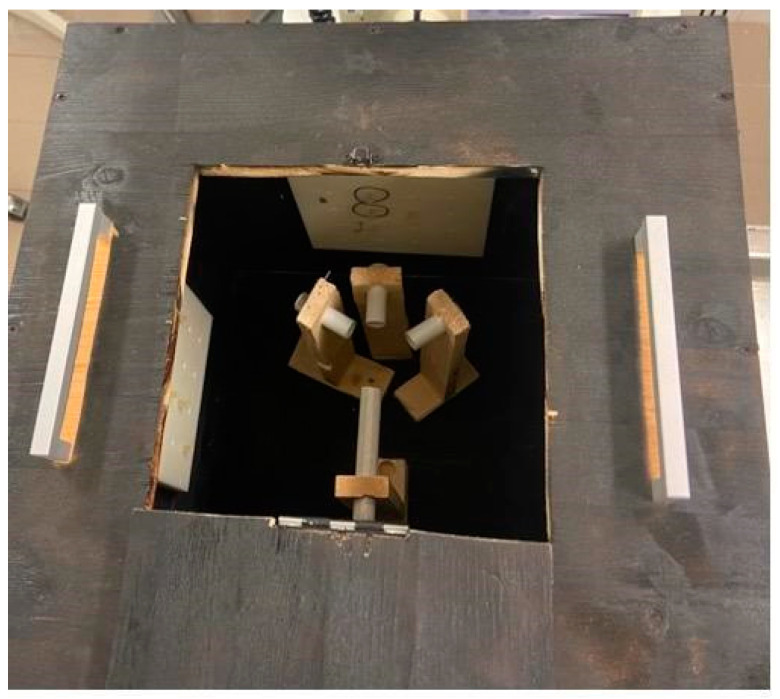
Ground view of the training box that shows the system of tubes used for navigation in each task.

**Figure 5:**
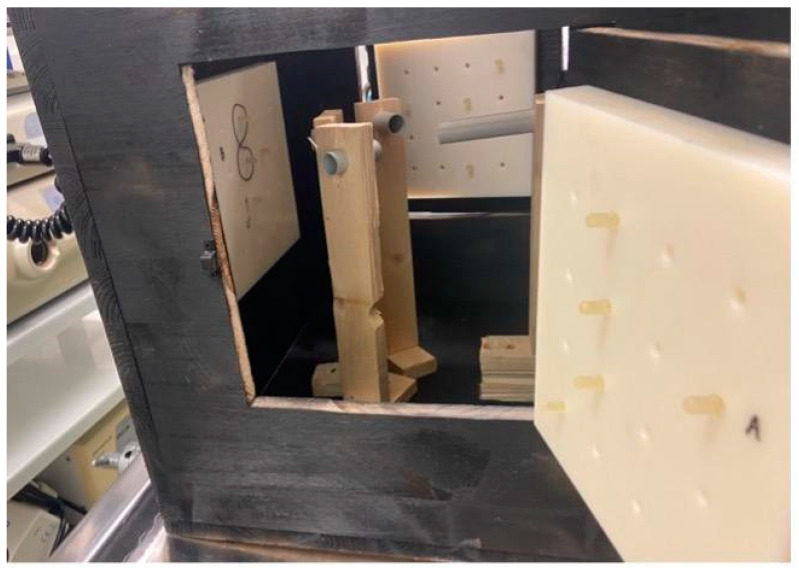
Lateral view of the training box through one of the side doors. On the right, a platform with pins is fitted on the door; a pathway for the endoscope with plastic tubes is visible inside the box.

In order to obtain the best possible results, we standardized the conditions for all participants. Our goal was to replicate conditions in the form of a training model that includes basic endoscopic movements, is straightforward and portable, and is user- and environmentally friendly. Our inspiration was founded in an article which described a special course box for endoscopists, with the intent to evaluate and measure their endoscopic abilities. Snare manipulation, loop solving, pinpoint accuracy, object moving, and forceps manipulation were the tasks at hand. Each participant was awarded points upon task completion, and additional points were given if the task was completed ahead of the designated time frame [4]. However, this assessment tool had a complicated design with many entry points and several types of mechanics. Our vision was to have a simplistic training box with one entry point and the possibility of multiple endoscopic tasks. We wanted it to be lightweight, portable, and durable. Through trial and error, we designed a cube-like box with one introduction point for the endoscope. Additionally, four doors were fitted on different sides. Inside the box, a pathway for the endoscope was constructed with a set of plastic tubes. The pathway allowed the endoscope to reach 3 different positions; each position ended as an endoscopic task which was fitted on the side door of the cube. A plate with pins and holes provided several options for different endoscopic tasks to be performed. Several repeated experiments were performed to ensure task completion. The building material for the box was primarily wood, the tube pathway was made from plastic, and plates with endoscopic tasks were manufactured with a 3D printer. To emulate real-life conditions, we painted the inside of the box black; hence, the endoscope was the only source of light inside the box. The layout of the training box is shown in Figure 3, Figure 4 and Figure 5.

**Figure 6:**
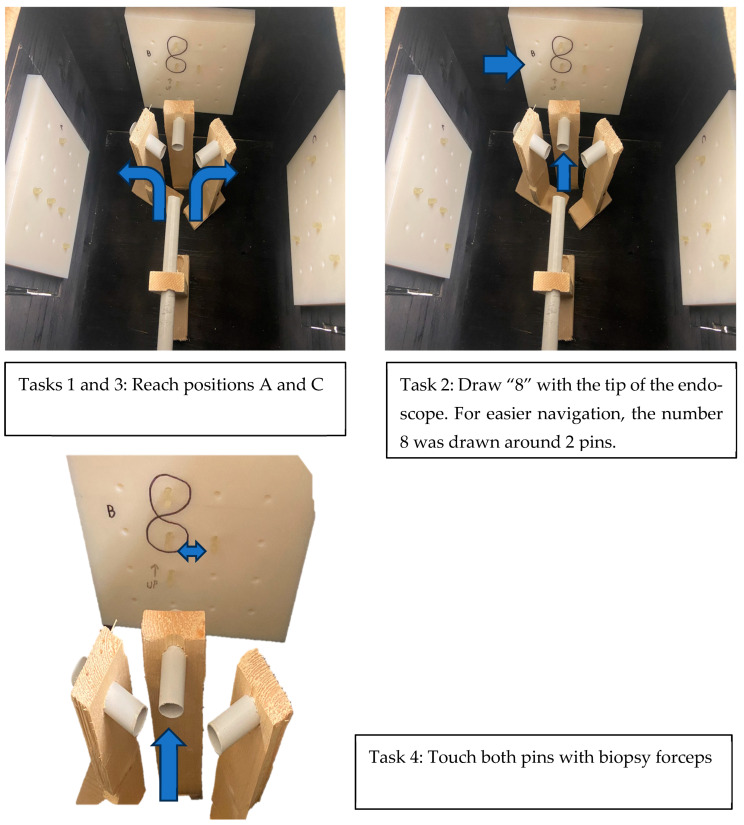
Task design.

The training box was designed as a training model to simulate everyday endoscopic maneuvering. Diagnostic endoscopy requires good maneuvering skills and good postural stability. In addition to these requirements, therapeutic endoscopy requires precise movements conducted during lengthier procedure times. We designed a box that can be used to simulate tasks performed in diagnostic and therapeutic procedures that require reaching certain positions and moving objects. Our objective was to emulate an endoscopic procedure, including insertion, maneuvering, reaching a certain target, and biopsy completion. The first task required a participant to reach task station “A” through the left tube. The second task required a participant to relocate the endoscope to the middle tube and draw the number eight three times between two pins. The third task required a participant to reach task station “C” through the correct set of tubes, and the fourth task consisted of moving an object (a rubber band) using forceps, from a pin to an adjacent pin. As the participants had to complete this without the assistance of a second person due to camera recording requirements, we modulated the fourth task. Each participant was required to insert endoscopic forceps into the endoscope, insert the endoscope into station “B” with 2 pins, and touch both pins with the forceps. After each task was completed, the participant was required to return the endoscope to the starting position. Each participant repeated the same set of maneuvers 5 times, always in a different order: first without using the holder and then while using the holder. In this way, we recorded a total of 20 captions without and 20 captions with the holder. The task design is shown in Figure 6.

### 2.4. Measurement Setup

A markerless motion capture system was used for the evaluation of participants’ body posture. With the use of a MoCap camera system, we recorded endoscopists while they were simulating endoscopic tasks using a specially designed endoscopic model.

**Figure 7:**
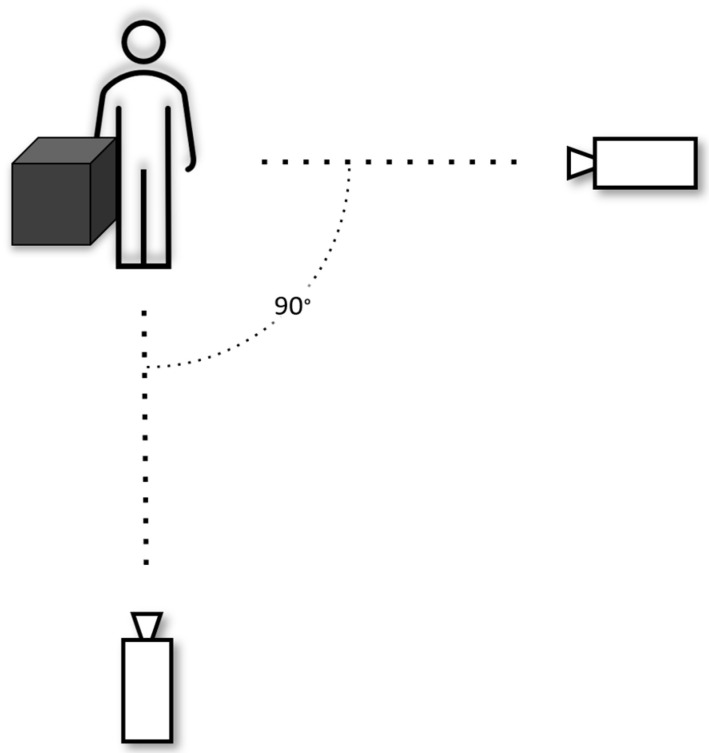
Experimental setup with 2 cameras.

**Figure 8:**
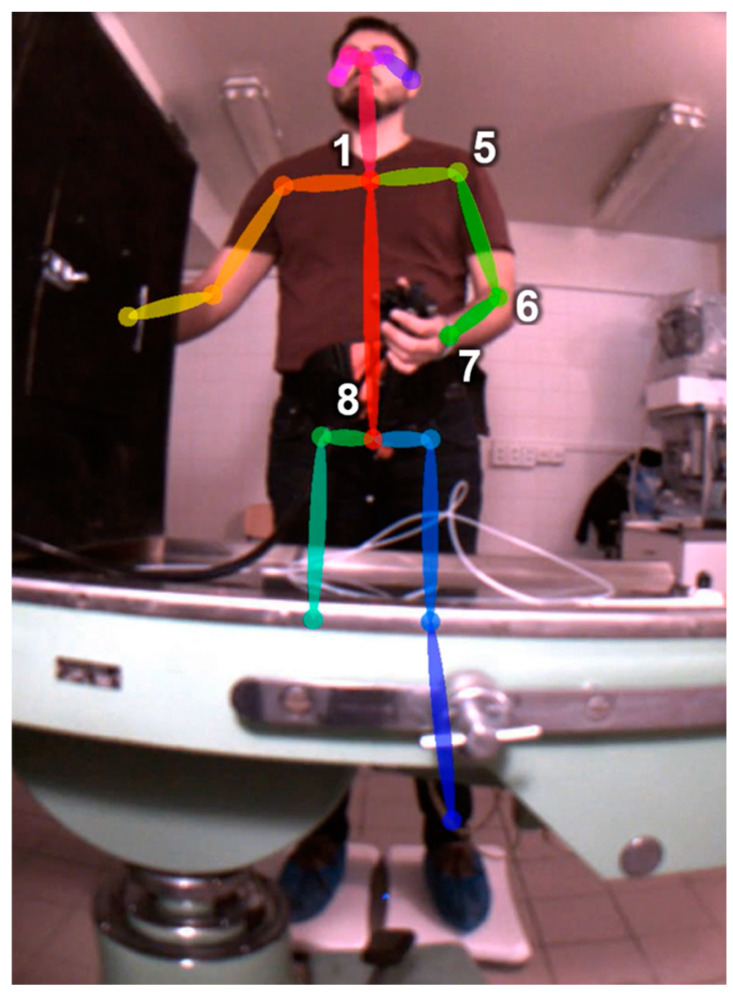
Evaluated keypoints (frontal view): 1 and 8 sagital body axis; 1, 5, 6, 7 left shoulder and elbow.

**Figure 9:**
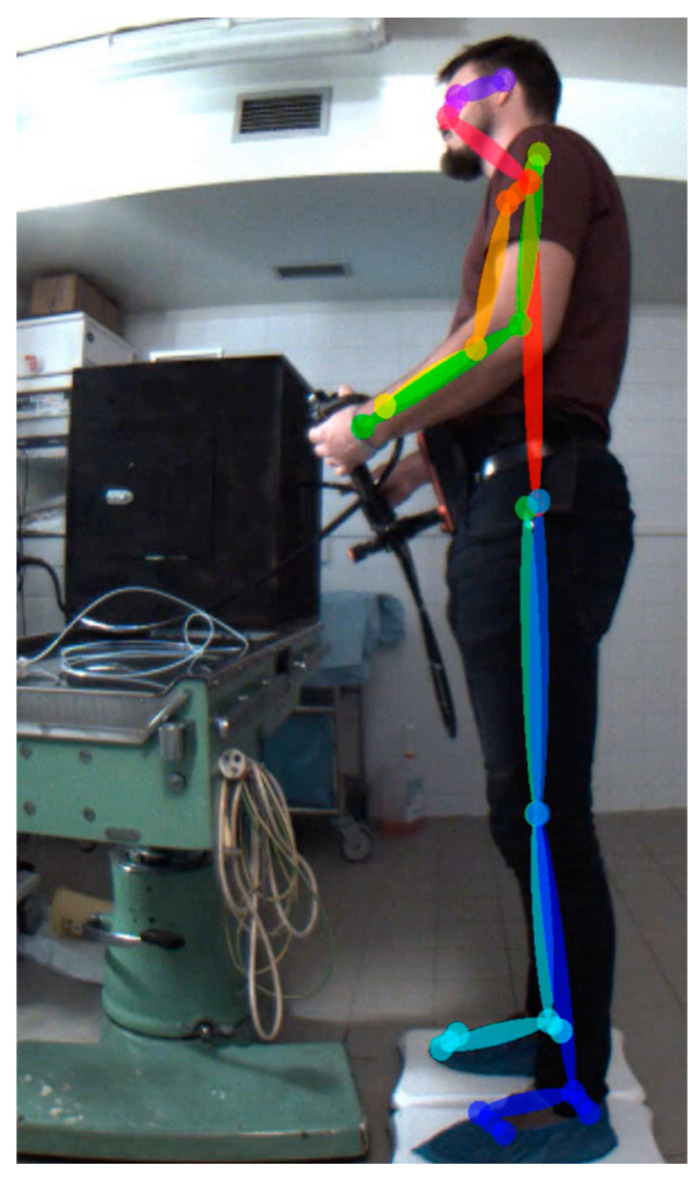
Evaluated keypoints (lateral view).

We used the adapted solution described in [5] in this study. This consisted of having the FLIR Blackfly 1.3 MPx RGB cameras positioned orthogonally in front of the subject (frontal view) or to the side (lateral view) of the subject’s non-dominant hand (see Figure 7, Figure 8 and Figure 9). The correct positioning of the cameras, including their orientations relative to each other and to the Earth’s coordinate system, was ensured by using a laser level and collimators aligned with the sensing chips of the cameras.

The cameras had a synchronized shutter and a frame rate of 25 fps. The recorded videos were consequently processed using the OpenPose version 1.7.0 (GPU release), which determined the x–y positions of individual keypoints and the confidence of its determination (Figure 8 and Figure 9) [5,6]. Confidence determines the level of predicted certainty, where 0 indicates an inability to detect a keypoint and 1 indicates a very high probability of correct detection. A value of 0.5 was chosen as the minimum confidence level for the analysis; however, during the measurements this value was rarely less than 0.6.

The time series of angles between the three selected keypoints, or between two keypoints and the vertical determined by the camera chip, was selected for further evaluation, specifically, the axial (keypoints 1 and 8), shoulder (keypoints 1, 5, and 6), and elbow (keypoints 5, 6, and 7) in the case of the frontal view and the elbow in the case of the lateral view. Regarding the other angles, either an influence by the holder is not expected or the necessary keypoints cannot be determined with sufficient confidence.

### 2.5. Statistical Evaluation

As a normal distribution of the data series was not expected, the following parameters were chosen for the analysis: median, interquartile range (IQR), total trajectory length (TL), and angular velocity (v). TL is defined as

$$TL=\sum_{i=1}^{N-1}\left|\alpha_{i}-\alpha_{i-1}\right|$$

where $\alpha_{0}...\alpha_{N-1}$ are angles defined by specified keypoints in individual frames and N is the total number of frames. v is defined as

v=TL/N∗fps

A non-parametric two-sided Wilcoxon rank-sum test with a 5% significance level was used to identify differences resulting from the use of the holder. All output data were analyzed using MATLAB R2024a. For the purpose of visualization, al_goodplot was employed [7] to provide an overall view of the data, as it creates a violin plot showing data distribution, mean value (star), median (black horizontal line), IQR (hourglass), and standard deviation (rectangle).

### 2.6. Rapid Upper Limb Assessment Tool (RULA)

The rapid upper limb assessment (RULA) tool [8] is an ergonomic-based workplace risk assessment tool that allows you to calculate the risk of musculoskeletal loading within the upper limbs and neck. RULA is easy and quick to use and does not require expensive equipment to complete. It comes in the form of a specially designed questionnaire, with the end score from 1 to 7, suggesting different actions or measures. In addition to our previous methods, we will also perform a RULA assessment with and without the holder.

## 3. Results

**Figure 10:**
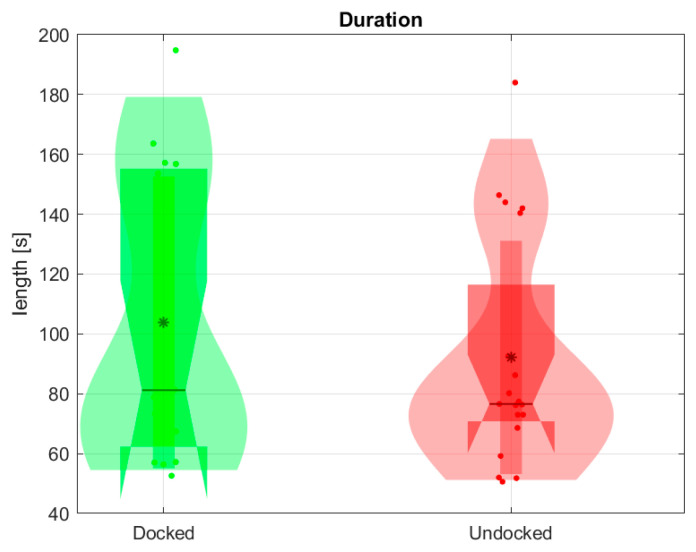
Session durations with (Docked) and without (Undocked) the holder. * is mean value.

Firstly, we compared the duration of simulation sessions that were performed with the belt holder (referred to as being “Docked”) and without the belt holder (referred to as being “Undocked”). The duration of each session is depicted in Figure 10; median session durations were 81 s for Docked sessions and 77 s for Undocked sessions. However, based on the findings from the Wilcoxon rank sum test, the holder did not have a statistically significant effect on session duration.

**Figure 11:**
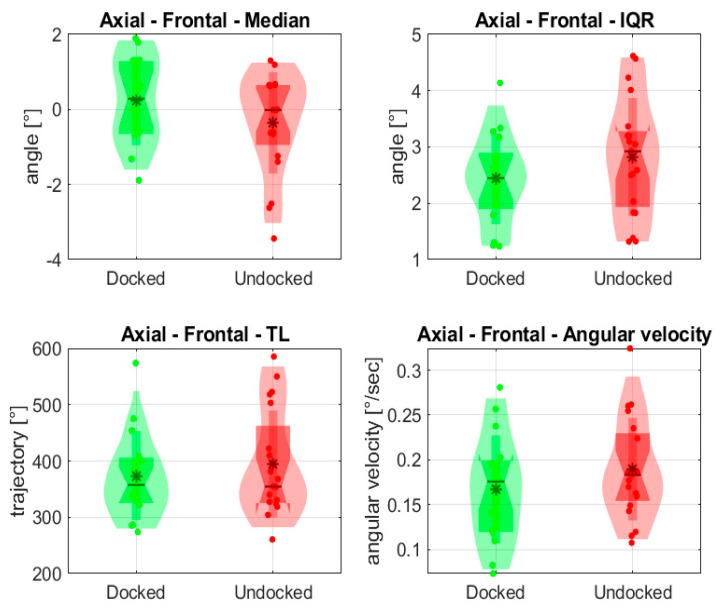
Evaluated statistical variables of the axial angle from the frontal view; * is mean value.

Variables evaluated from the frontal view are depicted In Figure 11.

**Figure 12:**
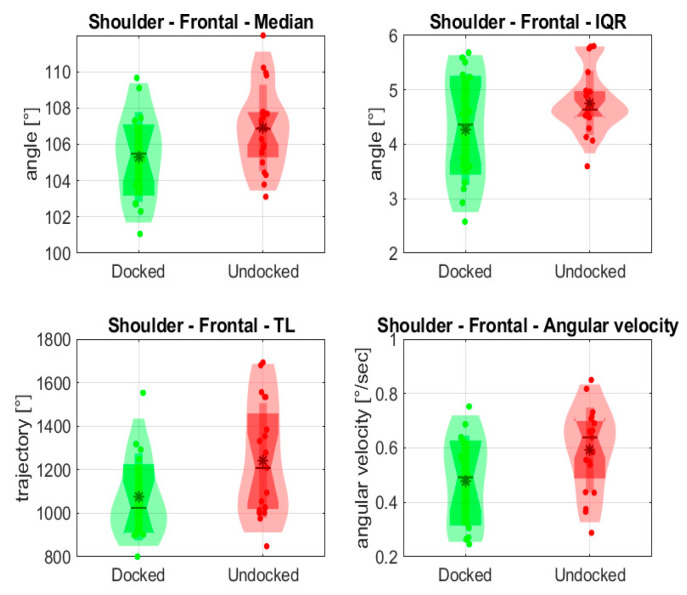
Evaluated statistical variables of shoulder angle from the frontal view. * is mean value.

In Figure 12, evaluated variables for the left shoulder are depicted. The Docked median angle was 105°, and the Undocked median angle was 107°; statistical analyses determined that these values were not significantly different (*p* = 0.053). The difference was observed in the case of the mean angular velocity (0.49°/s with the holder; 0.64°/s without it).

**Figure 13:**
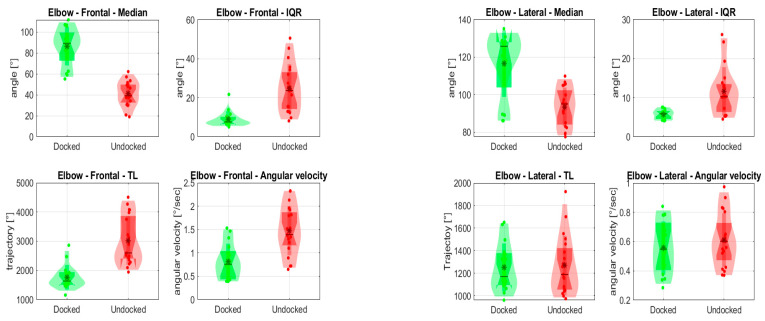
Evaluated statistical variables of elbow angle from the frontal and lateral view. * is mean value.

In Figure 13, the results for observed angles in the elbow are depicted (frontal and lateral view). Differences were observed for all the parameters studied from the frontal view; the use of the holder affects both the position (89.3°/39.2°) and interquartile range (7.6°/23.7°) of the elbow, as well as the total trajectory performed (1636/2600) and the mean value of the angular velocity (0.76/1.39). From the lateral view, a difference in position (125.8°/95.1°), interquartile range (5.9°/10.3°), total trajectory performed (1171/1189), and mean angular velocity (0.55/0.6) is observed.

Table 1 depicts the results of a two-sided Wilcoxon rank sum test of statistical parameters and angles monitored. For the frontal view, statistically significant differences are observed for angular velocity in the shoulder and for all observed parameters in the elbow. In the case of the lateral view, there are statistically significant differences in the median and IQR at the elbow.

The medians of the observed statistical parameters for all measurements with and without the holder are plotted in Table 2 and Table 3.

**Figure 14:**
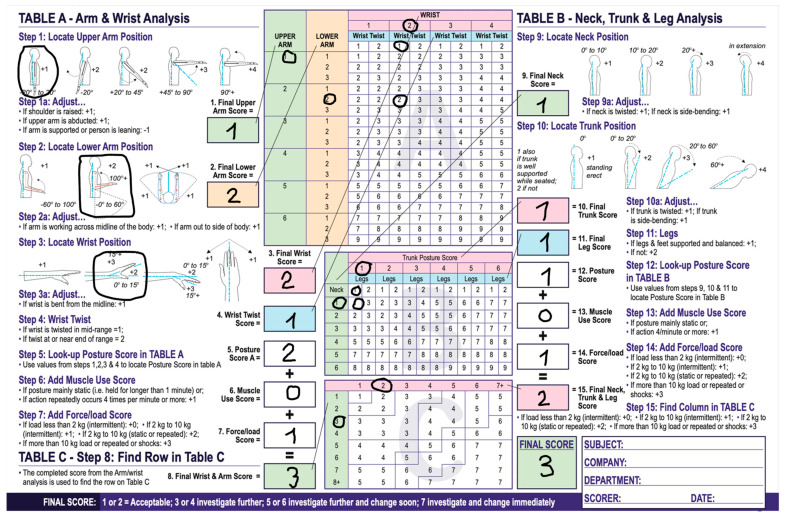
RULA score without the holder reached 3 points suggesting further investigation.

**Figure 15:**
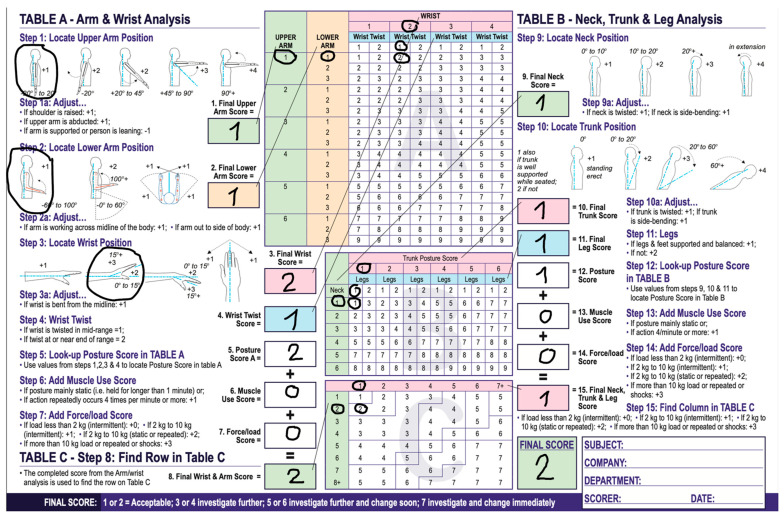
RULA score with the holder reached 2 points pointing to an ergonomically satisfying body and limb position.

In Figure 14 and Figure 15 RULA scores are presented firstly for an endoscopist holding the endoscope without the holder and secondly with the holder.

## 4. Discussion

Our study was designed as a natural continuation of a study using EMG measurements in which we were able to demonstrate that application of an endoscope-holding device can affect muscle loading and contribute to MSK injury prevention [3]. Alongside this, we assumed that the endoscope holder also affects body posture and body movement. We used an approach similar to those of other ergonomic studies by assessing body postures through video analysis [9].

To test the effects of using the endoscope holder on the positions of various body segments, we used an endoscopic training model from our previous EMG study [3]. However, certain changes had to be made. We slightly altered our task design due to video-recording requirements; in order to achieve the best possible recordings, a second person was not allowed in the room to serve as an assistant. Therefore, we adapted the fourth task in which an endoscopist would have to move objects with the endoscopic forceps so that each participant was only required to touch objects with them instead of moving them.

The study participants were highly trained endoscopists, for whom no additional training or education in the simulation tasks was needed. Our results demonstrated that no significant change (*p* > 0.05) in the duration of the simulation sessions resulted from using the endoscope-holding device.

Firstly, we looked at the frontal view and evaluated the body axis. Our results showed that using the endoscope-holding device did not cause a deviation in the body axis.

In evaluating the left shoulder we could not demonstrate a statistically significant difference in the median angle (*p* = 0.053); however, there was a noticeable difference in the angular velocity of 0.15°/s. This finding implies that a gradual or unhurried type of shoulder movement was used to complete the endoscopic tasks, which is supported by the similar movement trajectories and session durations observed for both Docked and Undocked sessions.

The most notable differences were observed in the frontal and lateral recordings of the left elbow. The use of an endoscope holder affected all observed parameters, predominantly in the frontal view. From the results obtained, we can assume that the holder allows the procedure to be performed with fewer movements of the elbow in the frontal plane, and that such movements are slower and spatially smaller in range than when a holder is not used. In the lateral plane, the holder has a significant effect on the position and interquartile range; however, total trajectory and angular speed are not affected. The above indicates the execution of finer movements.

Our findings give additional insight into the application of endoscope holders. With its belt-like position, the holder distributes the weight of the endoscope over the endoscopist’s body in addition to forcing the hands of the endoscopist into a more relaxed position (with a lower median angle of the left elbow). This finding correlates with the results of an EMG study [3], where there was a significant decrease in muscle load in the left forearm, biceps, and trapezius muscles as a direct effect of endoscope weight distribution to the endoscopist’s body due to the endoscope holder. These principles of improving body position and weight distribution are also described as methods to prevent MSK injuries in the workplace [10].

Furthermore, the left arm serves as a lever for the body. With the use of the holder, the left arm is in a more ergonomic position as a shorter lever in relation to the body; hence, there is less downforce and muscle load in the cervical spine as was demonstrated in the previous EMG study [3]. Pain in the hands, arms, and neck area were the main complaints of endoscopists [1]. Active and passive muscles become energetically depleted and overloaded after an undefined period of exercising a specific set of moves and shift to anaerobic metabolism. Fibrosis, atrophy, and shortening of the muscles are consequences of chronic overload. Impingement of adjacent structures can cause pain and movement disability [11].

Ergonomic calculations for the upper extremities can be made through rapid upper limb assessment (RULA) [8]. RULA is a well-established tool for ergonomic assessments that is used in occupations that involve the upper extremities. To perform RULA, observations are made of limb and body postures during specific tasks. The score is marked through a specific score sheet, where points are added or subtracted, depending on the limb and body position used to perform certain tasks. In general, the higher the RULA score, the greater the need for ergonomic intervention [12,13]. We performed the RULA with and without the holder. When using the holder, the final score was 2 which pointed to an ergonomically balanced position. Without the holder, when we used the RULA, the score was 3 points, suggesting that further investigation is needed. Compared to our results, a clear connection can be established. Fewer movements of the hand are made when using the belt-like holder, which results in a lower score in a RULA. Without the holder, there are more movements present, resulting in a higher RULA score. Hence, it can be deduced that there is an ergonomic benefit from using the holder.

Changes that are imposed by the use of the holder are minor in relation to traditional endoscopy. However, a note of caution is to be remembered: over time, small changes in body and limb positions result in big effects [10]. If one is to imagine the procedure volume and days spent exercising endoscopy in a certain way, then the overloading of a specific set of muscles would lead to an occupational injury and lower quality of life [14].

As this is an experimental research study, there are a few limitations that need to be taken into consideration. The study design demanded specific equipment and highly trained participants (experienced endoscopists). A low number of participants reduces the statistical weight of the results obtained. However, we obtained a consistent set of data with four participants, which would also probably be the case if we included more participants. A study using a larger sample would provide more statistically significant results.

Overall, with our research, we were not able to demonstrate that the use of an endoscope holder directly affects the postural stability of an endoscopist, and, above all, we could not effectively assess the effect of the device on the ergonomics and behavior of selected body segments. However, we were able to provide new information on limb movements during endoscopic procedures. Our findings express the need for further testing in a clinical environment in order to fully explore the extent of injury prevention related to the holder.

## 5. Conclusions

The prevention of MSK injuries in endoscopists during GI endoscopic procedures poses a challenge. Until now, only a set of basic ergonomic principles in the form of guidelines has been presented.

However, progress in this area is slow due to financial and organizational constraints. With the introduction of a novel ergonomic accessory for endoscopes, a step in the direction of preventing MSK injuries in GI endoscopy is possible. Through our studies, we have been able to demonstrate that we can help prevent MSK injuries related to GI endoscopy with ergonomic accessorise. We feel that the most crucial step would be to change the endoscope’s design and to change the process of endoscopic procedures to promote an injury-free work environment for all GI physicians [15].

## Figures and Tables

**Table 1:** The *p*-values of a two-sided Wilcoxon rank sum test of statistical parameters and angles monitored.

View	Angle	Median	IQR	TL	Angular Velocity [°/s]
Frontal	Axial	0.362	0.214	0.703	0.362
	Shoulder	0.053	0.18	0.057	0.049
	Elbow	<0.001	<0.001	<0.001	0.001
Lateral	Elbow	0.001	0.001	0.987	0.436

**Table 2:** Median of statistical parameters with the holder.

View	Angle	Median [°]	IQR [°]	TL [°]	Angular Velocity [°/s]
Frontal	Axial	0.29	2.5	358	0.18
	Shoulder	105	4.4	1025	0.49
	Elbow	89.3	7.6	1636	0.76
Lateral	Elbow	125.8	5.9	1171	0.55

**Table 3:** Median of statistical parameters without the holder.

View	Angle	Median [°]	IQR [°]	TL [°]	Angular Velocity [°/s]
Frontal	Axial	−0.01	2.9	355	0.18
	Shoulder	107	4.6	1208	0.64
	Elbow	39.2	23.7	2600	1.39
Lateral	Elbow	95.1	10.3	1189	0.6

## Data Availability

The raw data supporting the conclusions of this article will be made available by the authors on request.
